# Prevalence of Chronic Hepatitis B Virus Infection in Sierra Leone, 1997–2022: A Systematic Review and Meta-Analysis

**DOI:** 10.4269/ajtmh.22-0711

**Published:** 2023-05-22

**Authors:** George A. Yendewa, Gi-Ming Wang, Peter B. James, Samuel P. E. Massaquoi, Sahr A. Yendewa, Manal Ghazawi, Lawrence S. Babawo, Ponsiano Ocama, James B. W. Russell, Gibrilla F. Deen, Foday Sahr, Mustapha Kabba, Curtis Tatsuoka, Sulaiman Lakoh, Robert A. Salata

**Affiliations:** ^1^ Department of Medicine, Case Western Reserve University School of Medicine, Cleveland, Ohio;; ^2^ Division of Infectious Diseases and HIV Medicine, Case Western Reserve University School of Medicine, University Hospitals Cleveland Medical Center, Cleveland, Ohio;; ^3^ Johns Hopkins Bloomberg School of Public Health, Baltimore, Maryland;; ^4^ Case Comprehensive Cancer Center, Case Western Reserve University School of Medicine, Cleveland, Ohio;; ^5^ Faculty of Health, Southern Cross University, Lismore, Australia;; ^6^ Ministry of Health and Sanitation, Freetown, Sierra Leone;; ^7^ KnowHep Foundation, Freetown, Sierra Leone;; ^8^ Department of Nursing, School of Community Health Sciences, Njala University, Bo, Sierra Leone;; ^9^ Department of Medicine, College of Health Sciences, Makerere University, Kampala, Uganda;; ^10^ Department of Medicine, College of Medicine and Allied Health Sciences, University of Sierra Leone, Freetown, Sierra Leone;; ^11^ Connaught Hospital, University of Sierra Leone Teaching Hospitals Complex, Ministry of Health and Sanitation, Freetown, Sierra Leone;; ^12^ Centers for AIDS Research, Case Western Reserve University School of Medicine, Cleveland, Ohio

## Abstract

Hepatitis B virus (HBV) infection is a major public health problem in Sierra Leone, yet reliable estimates of cases are lacking. This study aimed to provide an estimate of the national prevalence of chronic HBV infection in the general population and select groups in Sierra Leone. We used the electronic databases PubMed/MEDLINE, Embase, Scopus, ScienceDirect, Web of Science, Google Scholar, and African Journals Online to systematically review articles reporting hepatitis B infection surface antigen seroprevalence estimates in Sierra Leone during 1997–2022. We estimated pooled HBV seroprevalence rates and assessed potential sources of heterogeneity. Of 546 publications screened, 22 studies with a total sample size of 107,186 people were included in the systematic review and meta-analysis. The pooled prevalence of chronic HBV infection was 13.0% (95% CI, 10.0–16.0) (*I*^2^ = 99%; *P*_heterogeneity_ < 0.01). During the study period, the HBV prevalence rates were as follows: 17.9% (95% CI, 6.7–39.8) before 2015, 13.3% (95% CI, 10.4–16.9) during 2015–2019, and 10.7% (95% CI, 7.5–14.9) during 2020–2022. The use of the 2020–2022 HBV prevalence estimates corresponded to 870,000 cases of chronic HBV infection (uncertainty interval, 610,000–1,213,000), or approximately one in nine people. The highest HBV seroprevalence estimates were among adolescents aged 10–17 years (17.0%; 95% CI, 8.8–30.5), Ebola survivors (36.8%; 95% CI, 26.2–48.8), people living with HIV (15.9%; 95% CI, 10.6–23.0), and those in the Northern Province (19.0%; 95% CI, 6.4–44.7) and Southern Province (19.7%; 95% CI, 10.9–32.8) regions. These findings may help inform national HBV program implementation in Sierra Leone.

## INTRODUCTION

Hepatitis B virus (HBV) is a double-stranded DNA virus of the Hepadnaviridae family that causes liver infection that can manifest as acute self-limiting hepatitis, fulminant liver failure, or chronic disease. Globally, an estimated 2 billion people—namely one-third of the world’s population—have been exposed to HBV, with 1.5 million new cases occurring annually and 296 million chronically infected to date.^1^^,^^2^ The prevalence of HBV infection varies based on geographic location, with sub-Saharan Africa (SSA) and the West Pacific region accounting for 79% of chronic cases of HBV.^1^ Despite the high global burden of the infection, current estimates suggest that only 10% of people living with HBV have been diagnosed.^1^^,^^2^ Undiagnosed and untreated HBV infection confers an elevated lifetime risk of liver-related complications including cirrhosis, hepatocellular carcinoma, and end-stage liver disease events, which collectively accounted for 820,000 deaths in 2019.^1^

Unlike the epidemic associated with HIV, viral hepatitis has comparatively received little public health policy focus until relatively recently. The Global Health Sector Strategy^3^ and the Sustainable Development Goals^4^ have recognized viral hepatitis as a neglected disease that is limiting the development of many low- and middle-income countries (LMICs) and have endorsed the elimination of HBV as a public health threat by the year 2030. Because there is currently no curative therapy for HBV given the persistence of the virus in hepatocytes, elimination efforts have focused on prevention via vaccination and treatment with antivirals. Vaccination is a potent public health strategy that is > 98% effective at preventing HBV infection,^1^ whereas treatment with nucleos(t)ide analogs can achieve durable suppression of viral replication and prevent liver-related morbidity and mortality.^5^ Despite the proven efficacy and cost-effectiveness of these strategies, many LMICs lack coordinated viral hepatitis prevention and control programs. In 2015, only three countries in the entire African region (viz., Algeria, Mauritania, and Senegal) had well-outlined national action plans for combating HBV.^6^

Sierra Leone is one of several countries in the West African region that is in the process of implementing a national policy for HBV infection. Accordingly, recent research efforts have focused on describing the characteristics of the HBV epidemic in the country. The majority of the studies—which have largely focused on blood donors, healthcare workers, pregnant women, and people living with HIV (PWH)—have reported HBV seroprevalence rates ≥ 8%,^7^ consistent with the WHO classification for hyperendemicity.^1^ Notwithstanding, there is limited understanding of the national prevalence of HBV infection in Sierra Leone because of insufficient surveillance systems. In hyperendemic settings such as Sierra Leone, HBV infection is acquired most frequently perinatally (by mother-to-child transmission) or horizontally via close contact during the early years of life.^1^^,^^8^^,^^9^ Despite this, HBV screening, vaccination, and treatment services remain limited in many such resource-constrained countries. Of note, HBV vaccination was introduced in Sierra Leone in 2007 and incorporated into the Expanded Program on Immunization (EPI) schedule for infants in 2009^10^; however, vaccination rates of all other groups including pregnant women, healthcare workers, and PWH have remained suboptimal.^10^

To ensure successful HBV program implementation in Sierra Leone and aid ongoing efforts toward the HBV elimination goals, it is essential to provide reliable estimations of the overall national HBV prevalence as well as identify groups that may be at high risk of infection for evidence-based policy planning and targeted public health interventions. We therefore performed a systematic review and meta-analysis of the available studies from all geographic regions of the country to estimate the prevalence of chronic HBV infection in Sierra Leone.

## MATERIALS AND METHODS

### Protocol registration.

The study was prospectively registered with the International Prospective Register of Systematic Reviews (registration number CRD42022337431). The conduct and reporting of the study were in accordance with Preferred Reporting Items for Systematic Reviews and Meta-Analyses (PRISMA) guidelines^11^ (Supplemental File 1).

### Outcomes of the analysis.

The primary outcome of interest was the national prevalence of chronic HBV infection in Sierra Leone, estimated as the pooled seroprevalence of the hepatitis B surface antigen (HBsAg) as detected by rapid diagnostic testing (RDT), ELISA, and other approved tests in primary studies conducted in Sierra Leone. Chronic HBV infection is defined as the persistence of HBsAg at least 6 months after acute infection. The secondary outcome of interest was the prevalence of chronic HBV in select populations in the subgroup analysis. We defined two population groups, categorized as 1) general populations, which included blood donors, healthcare workers, pregnant women seeking prenatal care, and other healthy people, and 2) special populations with a perceived higher risk of HBV infection, which consisted of PWH and Ebola virus disease (EVD) survivors.

### Country characteristics.

Sierra Leone is a country in West Africa with an estimated population of 8.14 million and a gross domestic product per capita of 516 U.S. dollars in 2021.^12^ Since 2017, the country has been divided into five administrative regions, namely the Northern, North West, Southern, and Eastern provinces and the Western Area. For the purposes of this study, we retained the historical (pre-2017) classification by aggregating the contemporary Northern and North West provinces as a single Northern region, the rationale being to simplify the process of determining study settings (Supplemental File 2). Freetown, the capital and largest city in Sierra Leone, is located in the Western Area and has an estimated population of 1.2 million.^10^ In recent years, Sierra Leone has faced a brutal civil war (1990–2001) and significant public health challenges, including parallel HIV and HBV epidemics and the West Africa Ebola epidemic (2014–2016), which have aligned to contribute to a fragile healthcare system.^13^ In 2016, there were an estimated 1.4 doctors, nurses, and midwives per 10,000 of the population, one of the lowest anywhere in the world.^14^ The latest Sierra Leone Demographic Health Survey (2019) reported an infant mortality rate of 75 per 1,000 live births, maternal mortality of 717 deaths per 100,000 live births, and adult mortality rate of 5.14 per 1,000.^15^ Despite these health indicators, the health expenditure per capita has remained low (i.e., 8.75% of the gross national product in 2020),^12^ and over 60% of healthcare costs are financed by end users via out-of-pocket payments.^16^

### Study design and search strategy.

We systematically searched the online electronic databases PubMed/MEDLINE, Embase, Scopus, ScienceDirect, Web of Science, Google Scholar, and African Journals Online for full-length texts of studies that assessed the seroprevalence of HBV in Serra Leone from inception to July 1, 2022. Our search terms included “hepatitis B,” “hepatitis B virus,” “hepatitis infection,” and “Sierra Leone” and were combined using the Boolean operators “OR” and “AND.” Additionally, we manually searched reference lists of studies to identify eligible articles.

### Inclusion and exclusion criteria.

We considered full-text articles of observational studies published in English that reported the seroprevalence of HBsAg conducted in Sierra Leone from database inception to July 1, 2022. Conference abstracts, case reports, case series, systematic reviews and meta-analyses, minireviews, editorials, commentaries, correspondence, studies that did not report a seroprevalence rate for HBsAg, studies with self-reported HBV infection status, and animal studies were not considered for inclusion. Where the full-text article of an otherwise eligible study was not available, the study was excluded. Additionally, we excluded studies conducted on Sierra Leonean-origin immigrant populations settled outside of the country.

### Study selection, screening, and data extraction.

Three authors (G. A. Y., P. B. J., and S. P. E. M.) independently screened the titles and abstracts of publications using the specified inclusion criteria and consulted another author (R. A. S.) in cases of disagreement. After reaching a consensus on the eligibility of the studies, all three authors (G. A. Y., P. B. J., and S. P. E. M.) independently extracted data from full-text articles and entered the information into a spreadsheet (Supplemental File 3). The following data were extracted: first author, year of publication, sampling year, study setting (region), study design, study population, sample size, sex, age, HBsAg prevalence, and diagnostic method used.

### Quality assessment.

Assessment of the methodological quality of the studies and risk of bias was undertaken by three authors (G. A. Y., P. B. J., and S. P. E. M.) using the Newcastle–Ottawa Scale (NOS) under the three domains of selection, comparability, and exposure.^17^ The NOS contains nine items with a total maximum possible quality score (QS) of 9. Studies with QS of 9–8 were rated as very high quality, QS 7–6 as high quality, QS 5–4 as moderate quality, and QS 3–0 as low quality. However, we decided a priori to include all eligible studies regardless of quality rating.

### Data analysis.

The meta-analysis was performed using the “metaphor” and “dmetar” packages of the statistical software R (version 4.0.4, Indianapolis, Indiana) by pooling data within a random-effects model using the DerSimonian and Laird estimator based on inverse variance weights. The magnitude of heterogeneity was reported as the heterogeneity (*I*^2^) index and its significance was assessed by the χ^2^ test and Cochrane’s *Q* statistic. The degree of heterogeneity was reported as minimal (*I*^2^ < 25%), moderate (25% ≤ *I*^2^ < 50%), and high (*I*^2^ ≥ 50%). Subgroup and sensitivity analyses were performed to account for differences in HBV seroprevalence between studies. For subgroup analysis, subjects were categorized based on age, sex, geographic region, HIV status, EVD survivorship, whether they were healthcare workers, pregnancy, year of study, and diagnostic method used. Publication bias was evaluated by both funnel plot asymmetry and Egger’s test. In all computations, differences were considered statistically significant at *P* value < 0.05.

## RESULTS

### Identification and selection of studies.

**Figure 1.**
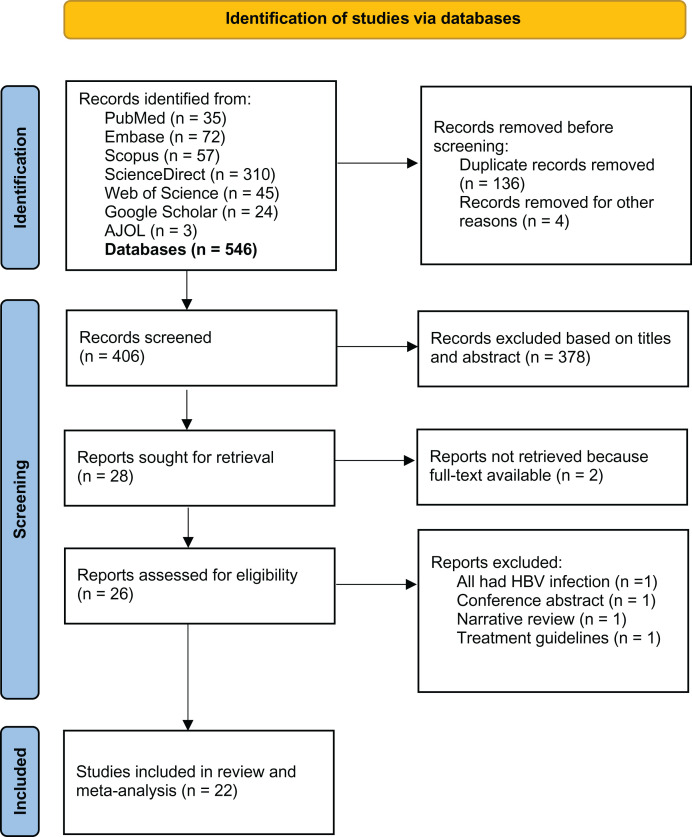
Flow diagram of a systematic review, Sierra Leone, 1997–2022. AJOL = African Journals Online; HBV = hepatitis B virus.

Figure 1 shows the PRISMA schematic flow of the records identified. A total of 546 published records were found by literature search using PubMed/MEDLINE, Embase, Scopus, ScienceDirect, Google Scholar, and African Journals Online. No additional studies were identified via a search of reference lists or other sources. After removing duplicates and screening studies based on title and abstract content, 28 articles were identified for full-text analysis. A total of 22 studies met the inclusion criteria and were included in the systematic review and meta-analysis.

### Characteristics of included studies.

Table 1 outlines the characteristics of the 22 studies included in the meta-analysis.^18^^–^^39^ The total sample size from the 22 studies was 107,186. The individual study sample sizes ranged from 142 to 43,163. Based on the NOS for quality assessment, six (27%) studies were rated as having very high quality (QS, 9–8), nine (41%) studies were of high quality (QS, 7–6), whereas the remaining seven (32%) were of moderate quality (QS, 5–4). The studies included spanned a period of 25 years (1997–2022), with the majority of studies (18; 82%) having been conducted after the incorporation of the HBV vaccine into the EPI schedule in Sierra Leone (i.e., 2010–2022).

**Table 1:** Summary characteristics of included studies on the prevalence of HBV in Sierra Leone, 1997–2022

First author	Publication year	Setting	Study design	Population	Sample size	Age (years)	Overall HBsAg prevalence	Method	Quality score
Breakwell et al.^18^	2022	Countrywide	Cross-sectional	Community	5,690	4 months to ≥ 18 years	3.9	RDT	9
Yendewa et al.^19^	2022	Western	Cross-sectional	HIV	174	15–75	15.5	ELISA	8
Ghazzawi et al.^20^	2022	Western	Cross-sectional	Pregnant women	394	16–44	7.9	ELISA	9
Wang et al.^21^	2021	Western	Cross-sectional	Febrile patients	142	2–44	8.5	ELISA	6
Bangura et al.^22^	2021	Eastern	Cross-sectional	Healthcare workers	632	Median 32	10.3	ICA	9
Yendewa et al.^23^	2021	Northern	Cross-sectional	HIV	183	2–9	10.8	CLIA	5
Mafopa et al.^24^	2020	Western	Cross-sectional	Healthcare workers, Ebola survivor	149	2–71	30.9	ELISA	8
Lawrence et al.^25^	2020	Eastern	Cross-sectional	Blood donors and patients	3,548	≤ 5 to ≥ 18	10.9	RDT	7
Kachimanga et al.^26^	2020	Western	Cross-sectional	Medical students, healthcare workers	157	Median 26	10.2	ELISA	6
Tognon et al.^27^	2020	Countrywide	Cross-sectional	Blood donors	29,713	14–93	10.8	RDT	9
Yendewa et al.^28^	2019	Western	Cross-sectional	HIV	211	16–64	21.7	CLIA	7
Yambasu et al.^29^	2018	Countrywide	Cross-sectional	Blood donors	16,807	Median 27	9.7	RDT	7
Massaquoi et al.^30^	2018	Western	Cross-sectional	Healthcare workers	447	N/A	8.7	LFA/ICA	6
Ngegba et al.^31^	2018	Western	Cross-sectional	Blood donors	43,163	18–55	15.2	RDT	5
Kangbai et al.^32^	2018	Southern	Cross-sectional	Young women	1,500	13–48	11.2	ICA	7
Qin et al.^33^	2018	Western	Cross-sectional	Healthcare workers	211	18–59	10	ELISA	6
Ansumana et al.^34^	2018	Southern	Cross-sectional	Febrile patients	860	5–50	13.7	ELISA	7
Zoker et al.^35^	2017	Southern	Cross-sectional	Adults	308	15–50	21.4	N/A	5
Koroma and Kangbai^36^	2014	Southern	Cross-sectional	Adolescents and young women	2,218	5 to ≥ 18	47.5	RDT	5
Adesida et al.^37^	2010	Western	Cross-sectional	Adults	198	N/A	21.7	RDT	4
Wurie et al.^38^	2005	Western	Cross-sectional	Pregnant women	302	16–40	6.2	ELISA	4
Torlesse et al.^39^	1997	Western	Cross-sectional	Pregnant women	179	N/A	11.3	ICA	5

CLIA = chemiluminescent immunoassay; HBsAg = hepatitis B infection surface antigen; HBV = hepatitis B virus; ICA = immunochromatography; LFA = laminar flow assay; N/A = not applicable; RDT = rapid diagnostic test.

The prevalence of HBsAg ranged from 3.9% to 47.5% in the individual primary studies. The age of study participants ranged from 4 months to 75 years. All geographic regions of Sierra Leone were represented, with 12 (55%) studies from the Western Area, four (18%) from the Southern Province, two (9%) from the Eastern Province, one (5%) from the Northern and North West provinces, two (9%) countrywide (Western, Eastern, Southern, Northern, and North West), and one (5%) interregional (Western, Southern, Northern, and North West). The majority of the subjects were from the Western Area (55%), followed by the Southern (18%), Eastern (13%), and Northern (13%) provinces. Blood donors accounted for the largest proportion of subjects (87%), followed by pregnant women (2.5%) and healthcare workers (1.4%). Children < 10 years old, PWH, EVD survivors, and others accounted for the remaining study participants (9%) (Table 1).

### Pooled prevalence of HBV.

**Figure 2.**
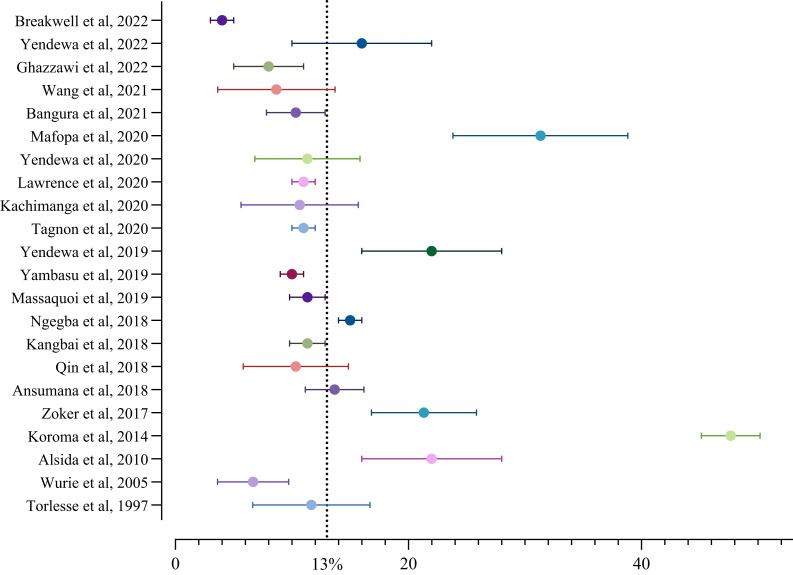
Forest plot of the pooled prevalence of hepatitis B virus in Sierra Leone, 1997–2022.

Using a random-effects model, the crude pooled prevalence of chronic HBV in Sierra Leone was 13.0% (95% CI, 10.0–16.0) in a pooled sample of 107,186 individuals (*I*^2^ = 99%; *P*_heterogeneity_ < 0.01) (Figure 2). In the sensitivity analysis, we removed the study with the highest seroprevalence of HBV (Koroma and Kangbai, 2014) to ensure the stability of the crude prevalence estimates. This yielded a sensitive HBV seroprevalence of 12.0% (95% CI, 10.0–14.0%) in a pooled sample of 104,968 individuals (*I*^2^ = 98%; *P*_heterogeneity_ < 0.001), which did not significantly differ from the crude estimates (Supplemental File 4).

Over the 25-year period covered by the primary studies, there appeared to be a trend toward lower HBV seroprevalence, although this decline did not meet statistical significance (*P* = 0.434): 17.9% (95% CI, 6.7–39.8) estimated from the studies conducted before 2015, 13.3% (95% CI, 10.4–16.9) during 2015–2019, and 10.7% (95% CI, 7.5–14.9) during 2020–2022 (Table 2). Using the 2020–2022 HBV prevalence (i.e., 10.7%) and the 2021 national population estimate of 8.14 million^12^ yielded an estimated 870,000 cases of chronic HBV infection nationwide (uncertainty interval, 610,000–1,213,000), corresponding to approximately one in nine people.

**Table 2:** Subgroup analysis of the HBV prevalence estimation in Sierra Leone, 1997–2022

Subgroup	Variable	Number of studies	Prevalence % (95% CI)	*I*^2^ %	*P* value
Age (years)	< 5	2	2.9 (0.6, 12.6)	96.0	< 0.001
	5–9	1	1.6 (1.1, 2.2)	N/A	
	10–17	1	17.0 (8.8, 30.5)	N/A	
	≥ 18	11	12.4 (9.9, 15.4)	97.3	
Sex	Male	17	10.5 (7.2, 15.0)	98.0	0.477
	Female	7	13.4 (7.4, 23.1)	99.4	
Geographic region	Western	13	11.2 (8.9, 14.1)	96.2	0.097
	Eastern	3	10.1 (9.1, 11.2)	66.5	
	Northern	2	19.0 (6.4, 44.7)	97.9	
	Southern	5	19.7 (10.9, 32.8)	99.6	
Study population	HIV	3	15.9 (10.6, 23.1)	76.1	0.320
	Ebola survivors	1	36.8 (26.2, 48.8)	N/A	< 0.001
	Healthcare workers	4	11.9 (7.7, 18.0)	79.8	0.632
	Pregnant women	4	9.7 (6.3, 14.6)	68.6	0.210
Diagnostic test	ELISA	8	11.8 (8.1, 16.8)	89.3	0.867
	RDT	7	13.9 (7.4, 24.8)	99.8	
	Others	7	13.0 (9.8, 17.2)	87.8	
Year of study (overall)	≤ 2014	4	17.9 (6.7, 39.8)	98.6	0.434
	2015–2019	8	13.3 (10.4, 16.9)	98.0	
	≥ 2020	10	10.7 (7.5, 14.9)	97.1	
Year of study (age ≥ 18 years)†	≤ 2014	3	11.6 (4.7, 26.0)	97.9	0.843
	2015–2019	4	13.5 (9.2, 19.4)	99.1	
	≥ 2020	8	11.9 (9.2, 15.2)	78.1	

ELISA = enzyme-linked immnosorbent assay; HBV = hepatitis B virus; N/A = not applicable; RDT = rapid diagnostic test.

† All the years of study with age < 18 years are 2020 or after.

### Subgroup analysis.

**Figure 3.**
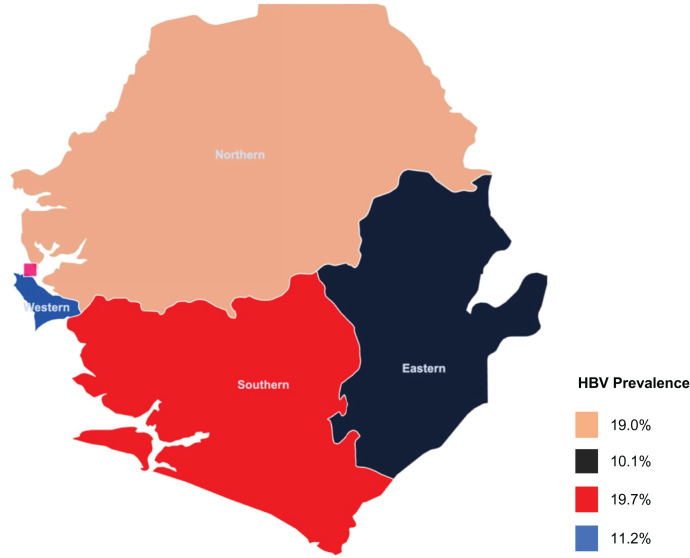
Map of Sierra Leone with the estimated HBV prevalence by region. HBV = hepatitis B virus.

Based on the studies that provided age-specific estimates, adolescents aged 10–17 years had the highest HBV seroprevalence (17.0%; 95% CI, 8.8–30.5), followed by adults aged ≥ 18 years (12.4%; 95% CI, 9.9–15.4) (Table 2). Children aged 5–9 years had the lowest HBV seroprevalence rates (1.6%; 95% CI, 1.1–2.2), whereas those younger than 5 years had an HBV seroprevalence of 2.9% (95% CI, 0.6–12.6). The HBV seroprevalence was 10.5% (95% CI, 7.2–15.0) among males and 13.4% (95% CI, 7.4–23.1) among females. The Northern (19.0%; 95% CI, 6.4–44.7) and Southern (19.7%; 95% CI, 10.9–32.8) regions had higher HBV seroprevalence rates compared with the Western Area (11.2%; 95% CI, 8.9–14.1) and Eastern Province (10.1%; 95% CI, 9.1–11.2) (Figure 3).

Regarding the general populations, the HBV seroprevalence among healthcare workers was 11.9% (95% CI, 7.7–18.0), whereas pregnant women seeking prenatal care had an HBV seroprevalence of 9.7% (95% CI, 7.7–14.6). Within the two special population groups, EVD survivors had a higher HBV seroprevalence (36.8%; 95% CI, 26.2–48.8), whereas HIV-infected people had an HBV seroprevalence estimate of 15.9% (95% CI, 10.6–23.1). Of the testing methods used, RDT accounted for the highest seroprevalence rates (13.9%; 95% CI, 7.4–24.8) versus ELISA (11.8%; 95% CI, 8.1–16.8) and other diagnostic methods (13%; 95% CI, 9.9–17.2).

To assess the potential impact of HBV vaccination on the declining HBV prevalence, participants aged < 18 years (i.e., vaccinated population) were moved from the analysis. The HBV prevalence estimates among adults aged ≥ 18 years remained stable across study periods, as follows: 11.6% versus 13.5% versus 11.9% (*P* = 0.843) during the periods before and during 2014 versus 2015–2019 versus during and after 2020, respectively (Table 2). Thus, the prevalence of HBV remained high and stable among the adult population, who therefore are at risk of developing liver-related complications including cirrhosis and hepatocellular carcinoma.

Overall, heterogeneity remained high among the studies, with the sources of heterogeneity significantly accounted for by age (*P*_difference_ < 0.001) and EVD survivorship (*P*_difference_ < 0.001). Geographic regions contributed substantially to HBV seroprevalence heterogeneity but did not attain statistical significance (*P*_difference_ < 0.097) (Table 2).

### Publication bias.

**Figure 4.**
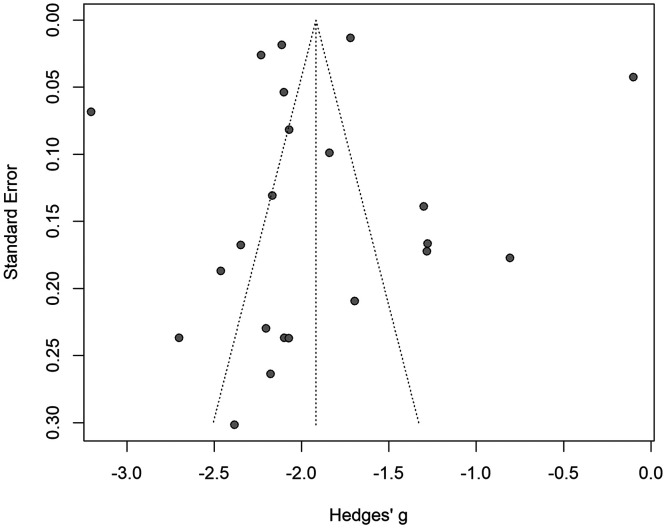
Funnel plot of the prevalence of hepatitis B virus estimation in Sierra Leone, 1997–2022.

Figure 4 shows the funnel plot. The Egger’s regression did not show asymmetry (*P* = 0.892), indicating no evidence of publication bias.

## DISCUSSION

This is the first systematic review and meta-analysis to estimate the prevalence of chronic HBV infection in Sierra Leone. The overall goal was to synthesize evidence to help inform ongoing efforts seeking to combat the HBV epidemic in the country. Data were pooled from general and special (high-risk) populations and all geographic regions were represented. The crude pooled prevalence of chronic HBV was 13%, which did not significantly differ from the 12% estimate obtained in the sensitivity analysis. Based on 2021 population estimates, about 870,000 people or one in nine Sierra Leoneans were estimated to have chronic HBV infection. Most (68%) of the primary studies included in the analysis were rated as having high methodological quality, suggesting a reliable estimate. Our findings are consistent with recent data from Ghana,^40^ Nigeria,^41^ Cameroon,^42^ and other countries in West Africa.^43^

Over the 25-year period covered by the study, the HBV prevalence did not decline significantly in Sierra Leone. This could be explained in part by the fact that HBV vaccination, safer healthcare practices (e.g., screening of blood for transfusion), and improved socioeconomic conditions have not been evenly accessible across different groups.^44^^,^^45^ For instance, according to UNICEF estimates, the HBV vaccination coverage for infants in Sierra Leone was 92% in 2020, surpassing the estimated global coverage of 80%.^46^ This was reflected in the low HBV seroprevalence observed among vaccinated children younger than 5 years (2.9%) and children aged 5–9 years (1.6%) in the subgroup analysis. On the other hand, vaccination efforts have not benefited other groups to the same extent (i.e., adults, pregnant women, healthcare workers, and PWH), with studies reporting vaccination rates ranging from 1% to 4.3% among these groups in Sierra Leone.^18^^,^^24^^,^^28^^,^^31^ Consequently, as shown in the subgroup analysis, the HBV prevalence rate has not declined significantly among adults, who continue to pose a public health risk to susceptible individuals in the population via horizontal modes of HBV transmission. Thus, directing targeted interventions at these groups such as HBV screening, vaccination, and treatment will be critical to achieving the HBV elimination goals in Sierra Leone.

We did not find significant differences in HBV seroprevalence based on sex, pregnancy status, being a healthcare worker, or testing method. Considering sex differences, our findings are in contrast with studies that have documented a higher prevalence of chronic HBV infection among males compared with females regardless of geographic region.^47^^–^^49^ Additionally, these sex disparities persist in the incidence and clinical outcomes of liver-related morbidity including hepatocellular carcinoma, with males more adversely impacted.^50^ Reasons that have been suggested for these differences include a preponderance of traditional risk factors among males (e.g., smoking, alcohol use, type 2 diabetes, nonalcoholic fatty liver disease/nonalcoholic steatohepatitis)^51^^,^^52^ and biological factors including higher HBV DNA levels in males^53^ and the differential effects of sex hormones on carcinogenesis.^54^ The lack of a sex differential in HBV prevalence in favor of males in our study could be accounted for in part by sampling bias inherent in the primary studies that were included in the analysis.

The HBV prevalence among adolescents aged 10–17 years was 17%, which was higher than in any other age category. Data on the prevalence and impact of HBV infection on adolescents are scarce; however, recent studies from West Africa and elsewhere suggest that adolescents and young adults have received little attention in the global elimination efforts.^55^ From a public health perspective, this group may pose a significant risk of onward transmission in the general population.^55^ A meta-analysis by Abesig et al.^56^ estimated a high HBV prevalence (14.3%) among adolescents in Ghana (2015–2019) compared with a prevalence of 8.36% among adults. In a 2018 study from a clinic screening for sexually transmitted infections (STIs) in Nigeria, Nejo et al.^57^ found a low HBV seroprevalence among adolescents (1.9%); however, the risk of HBV was 9-fold higher (HBV seroprevalence, 17%) in sexually actively adolescents. A plausible explanation for the disproportionately high impact of HBV on adolescents and young adults in SSA is that this group tends to have a high burden of risk factors that increases susceptibility to horizontal transmission of HBV and other STIs (notably HIV), including intravenous drug use, multiple sexual partners, tattooing and other body scarification procedures, and insufficient knowledge of disease processes due to cultural norms that restrict discussions around sexual practices.^55^^,^^56^^,^^58^^,^^59^ Others have noted that in endemic settings in SSA, most adolescents with chronic HBV infection are overlooked because they predominantly present in the immunotolerant phase of infection, which is characterized by slow clearance of HBsAg and high rates of viral replication without evidence of hepatic inflammation, and therefore rarely require treatment.^55^ This highlights the need for further research into the prevention, screening, and treatment strategies targeted at this population.

Our study uncovered regional disparities in HBV seroprevalence in Sierra Leone. The Northern and Southern regions had similarly high HBV seroprevalence rates (19% and 19.7%, respectively), almost double the rates in the Western Area (11.2%) and Eastern region (10.1%). The reasons for these regional variations are unclear but may include disparities in prenatal screening, uptake of vaccination, and availability of treatment services. Other barriers hampering HBV control efforts that have been documented in SSA include low socioeconomic status, poor knowledge of HBV, and low health literacy in general.^2^ Emerging research also suggests that similar to HIV, HBV-related stigma is pervasive in communities across SSA and may be influencing health-seeking behaviors, but this phenomenon is poorly understood.^60^ These factors need to be further explored because understanding the reasons behind these regional differences may be crucial to designing evidence-based healthcare-strengthening strategies to effectively tackle the HBV epidemic in the country.

We further observed a higher HBV seroprevalence among HIV-infected individuals (15.9%). HIV is a well-recognized risk factor for HBV infection and vice versa, due to shared risk factors and routes of transmission.^61^^,^^62^ A prominent feature of the HIV epidemic in West Africa is its intersection with HBV, which is a major driver of disease progression and outcomes in this region.^16^ A recent meta-analysis by Platt et al.^63^ estimated a global HBV seroprevalence of 7.6% among PWH. The highest HIV/HBV coinfection prevalence was observed in West and Central Africa at 16.4%, more than 2-fold higher than the global prevalence rate,^63^ which is consistent with our findings. The consequences of HIV/HBV coinfection have been well documented and include faster progression to AIDS, liver cirrhosis, and hepatocellular carcinoma.^61^^,^^62^ Thus, in HBV-hyperendemic countries such as Sierra Leone, successfully tackling the HIV epidemic will also require incorporating parallel efforts aimed at simultaneously combating HBV to meet the 2030 global HIV and HBV elimination targets. This warrants greater integration of HIV and HBV services, especially in West Africa, which is disproportionately affected by the HBV epidemic.

The prevalence of chronic HBV infection among EVD survivors was high at 36.8%. This estimate was from a single study by Mafopa et al.,^24^ which makes it difficult to draw generalizable inferences. The West African Ebola epidemic of 2014–2016 was a major public health crisis that resulted in 30,000 symptomatic cases and over 11,000 EBV-related deaths in Sierra Leone, Guinea, and Liberia.^64^ Despite the large number of EVD survivors, no studies to date have explored the potential interactions between Ebola virus and endemic viruses such as HBV, which are cocirculating in the general population in West Africa. However, it is well known that Ebola virus infection is accompanied by intense immune activation. In addition to the robust host innate immune response that characterizes acute Ebola virus infection via interleukin (IL)-1 beta, IL-6, tumor necrosis factor alpha, macrophage chemotactic protein 1, and other proinflammatory mediators,^65^^,^^66^ dysregulation of adaptive immunity (specifically CD4+ and CD8+ T-cell responses) has also been reported in fatal disease as well as among EVD survivors.^65^^,^^66^ It is plausible that in HBV-endemic settings such as Sierra Leone with large numbers of chronic HBV carriers (i.e., HBsAg−, anti-HBV core antibody positive), EVD survivors who are also HBV carriers may experience reverse seroconversion (i.e., HBsAg+, HBV DNA+) in the setting of a perturbed immune system,^67^ which could partly explain our findings. More research is needed to further investigate the potential interactions among Ebola virus, HBV, and other endemic viruses and the implications of such interactions for disease pathogenesis and outcomes in this setting.

Our study had several methodological limitations, most of which affect the generalizability of our findings. First, most of the studies relied on convenience sampling and a cross-sectional design, compromising the representativeness of the sampled population. Second, blood donors made up the majority (about 87%) of the study population. This population typically consists of young and otherwise healthy adults and excludes high-risk or vulnerable populations. Third, although all geographic regions were represented in the analysis, there was an imbalance in the number of studies across geographic regions, with the majority of studies having been conducted in the Western Area. Additionally, studies used different diagnostic methods with varying performance characteristics over the study period, which could have affected prevalence estimates. Despite these limitations, this meta-analysis is the first to provide a nationwide estimate of the prevalence of chronic HBV infection in Sierra Leone and will help inform public health policy toward the 2030 HBV global elimination goals.

## CONCLUSION

In conclusion, we observed a high prevalence (13%) and burden of chronic HBV infection in Sierra Leone four decades after the first HBV vaccine was produced, despite a progressive downward trend in seroprevalence in recent years. Adolescents, Ebola survivorship, PWH, and those residing in the Northern and Southern regions were major predisposing factors to having chronic HBV infection in this setting. These findings necessitate the urgent implementation of national HBV prevention and control programs in Sierra Leone to meet the 2030 viral hepatitis elimination goals.

## Supplemental Materials


Supplemental materials

Supplemental materials
